# An Animal Model with a Cardiomyocyte-Specific Deletion of Estrogen Receptor Alpha: Functional, Metabolic, and Differential Network Analysis

**DOI:** 10.1371/journal.pone.0101900

**Published:** 2014-07-07

**Authors:** Sriram Devanathan, Timothy Whitehead, George G. Schweitzer, Nicole Fettig, Attila Kovacs, Kenneth S. Korach, Brian N. Finck, Kooresh I. Shoghi

**Affiliations:** 1 Department of Radiology, Washington University in St. Louis, Saint Louis, Missouri, United States of America; 2 Division of Geriatrics and Nutritional Science, Department of Medicine, Washington University in St. Louis, Saint Louis, Missouri, United States of America; 3 Center for Cardiovascular Research, Department of Medicine, Washington University in St. Louis, Saint Louis, Missouri, United States of America; 4 Laboratory of Reproductive and Developmental Toxicology, Receptor Biology Section, National Institute of Environmental Health Sciences, National Institutes of Health, Research Triangle Park, North Carolina, United States of America; 5 Department of Biomedical Engineering, Washington University in St. Louis, Saint Louis, Missouri, United States of America; 6 Division of Biology and Biomedical Sciences, Washington University in St. Louis, Saint Louis, Missouri, United States of America; INRA, France

## Abstract

Estrogen exerts diverse biological effects in multiple tissues in both animals and humans. Much of the accumulated knowledge on the role of estrogen receptor (ER) in the heart has been obtained from studies using ovariectomized mice, whole body ER gene knock-out animal models, *ex vivo* heart studies, or from isolated cardiac myocytes. In light of the wide systemic influence of ER signaling in regulating a host of biological functions in multiple tissues, it is difficult to infer the direct role of ER on the heart. Therefore, we developed a mouse model with a cardiomyocyte-specific deletion of the ERα allele (cs-ERα^−/−^). Male and female cs-ERα^−/−^ mice with age/sex-matched wild type controls were examined for differences in cardiac structure and function by echocardiogram and differential gene expression microarray analysis. Our study revealed sex-differences in structural parameters in the hearts of cs-ERα^−/−^ mice, with minimal functional differences. Analysis of microarray data revealed differential variations in the expression of 208 genes affecting multiple transcriptional networks. Furthermore, we report sex-specific differences in the expression of 56 genes. Overall, we developed a mouse model with cardiac-specific deletion of ERα to characterize the role of ERα in the heart independent of systemic effects. Our results suggest that ERα is involved in controlling the expression of diverse genes and networks in the cardiomyocyte in a sex-dependent manner.

## Introduction

Estrogen receptors (ER) are involved in multiple biological processes in a variety of tissues including the cardiovascular system, adipose tissue, and sex organs [1]. Generally, the actions of ERs are triggered upon binding of estrogens such as 17-β estradiol (E2), the predominant active form of estrogen [2]. Upon ligand binding, ERs mediate their response by two possible modes of action: genomic, which elicits a slow response, and non-genomic, which induces a rapid response. Genomic activation of ERs initiate transcription factors either by direct DNA interaction through estrogen response elements (ERE) or through ER-DNA indirect interactions by tethering with known transcription factors bound to the DNA [3]. The non-genomic actions, on the other hand, are mostly membrane-initiated and involve signaling cascades, such as the mitogen-activated protein kinase (MAPK) pathway, the cyclic adenosine mono-phosphate/protein kinase A (cAMP/PKA) pathway, or the endothelial nitric oxide synthase (eNOS) pathway [4]–[6].

ERs have been implicated in several pathophysiological conditions including diabetes and obesity with implications for cardiovascular disease [1], [7]. In the heart, cardiomyocytes express both sub-types of ER, ERα and ERβ, with significantly higher levels of ERα [8]–[10]. Mice with whole-body deletion of ERα have been shown to exhibit altered cardiac substrate preference, particularly uptake and maintenance of glucose in the heart [11]. Indeed, whole-body ERα knockout mice are obese and insulin resistant [12]–[14] and exhibit diminished rates of fatty acid (FA) oxidation in skeletal muscle [13]. ERα is also thought to possess cardio-protective properties, due to its ability to up-regulate expression of ApoE, an apolipoprotein that increases clearance of low density lipoproteins (LDL) from circulation [15], [16]. Similarly, ERβ has been reported to mediate sex-differences in ischemia/reperfusion injury [17], [18] as well as protect against left-ventricular hypertrophy (LVH) in females [19]. Interestingly, ERα and ERβ have also been reported to differentially modulate the expression of inflammatory markers, in particular that of inducible nitric oxide synthase (iNOS) [20]. Nevertheless, to date, the studies describing the role of ERs on the cardiovascular system were based on whole-body ER knockouts or on the effects of ER ligands, such as estrogen, on the heart, but with the caveat that such ER ligands also affect peripheral tissues. In light of the wide influence of ER signaling in regulating physiologic functions in multiple tissues, including systemic energy homeostasis, and considering that peripheral substrates can induce cardio-metabolic remodeling, it is difficult to infer the direct role of ER on the heart using whole-body KO models.

As a first step in characterizing the role of ERs in the heart, independent of systemic effects of ERs, we have generated a mouse model with a cardiomyocyte-specific deletion of ERα (cs- ERα^−/−^. The availability of mice with cs-ERα^−/−^ will enable, for the first time, to investigate the role of ERα in cardiac tissue independent of peripheral effects. In addition, since ERs are important therapeutic targets, development of *in vivo* models of selective modulation (such as cardiac specific deletion) will enable better understanding of ER effects in specific tissue [21], [22]. To that end, we characterized basal cardiac structure and function, and performed gene expression microarray profiling to determine differentially affected networks and pathways in male and female mice. Statistically validated results from the microarray analyses were used for pathway analysis, with emphasis on transcriptional factors and receptor networks (for deciphering genomic actions of ERα). Our findings indicate that cs- ERα^−/−^ manifests significant variations in the expression profile of genes involved in metabolism, cell growth and differentiation, muscle architecture, and relaxation. Finally, we delineate sex differences associated with the absence of ERα in the heart and identify key transcriptional/receptor hubs that are involved in ERα mediated regulation/signaling in cardiac tissue.

## Materials and Methods

All experiments were conducted according to a protocol approved by the animal experiment committee at Washington University School of Medicine in Saint Louis (IACUC Animal Welfare Assurance # A-3381-01) and in accordance to ‘Principles of laboratory animal care’ (NIH pub no. 85–23, revised 1985; http://grants1.nih.gov/grants/olaw/references/phspol.htm). Animals were housed in AAALAC-Accredited animal facility overseen by experienced veterinary personnel and animal care staff. Animals were euthanized via carbon dioxide inhalation, and all efforts were made to minimize suffering.

### Generation of cardiomyocyte specific ERα knockout mice

**Figure 1:**
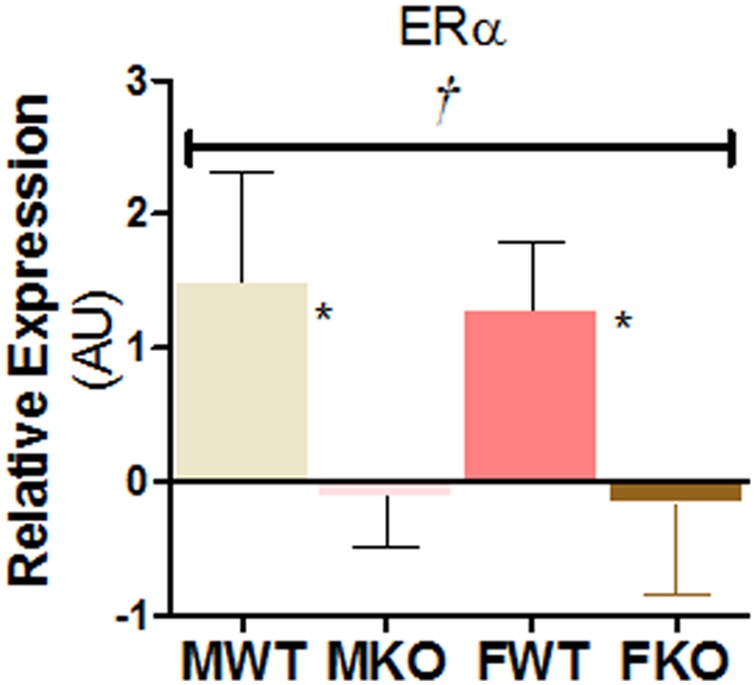
Extent of ERα knockout in the cardiac tissue: RNA from N = 3/group mice were isolated and expression analyzed using primetime assay primers from IDT DNA (Iowa). † represents genotype significance, Two-Way ANOVA and * indicates significant difference within sex (Contrast Analysis by Tumhane and Dunlop Method [37]). P<0.05 was considered significant for both.

Mice with cardiomyocyte-specific ERα deficiency were generated by crossing mice with Exon 3-floxed ERα alleles obtained from NIEHS [23] with mice expressing Cre recombinase in a cardiomyocyte specific manner (α-MHC-Cre). Mouse tail digest was used for genotyping. PCR screening was performed for Cre recombinase using the forward primer CGGTCAACGTGCAAAACAGGCTCTA and reverse primer CTTCCAGGGCGCGAGTTGA TAGC. The expression of ERα in cardiac tissue of the knockout mice were quantitatively measured by qRT-PCR using ERα Specific Primetime Assay primers (IDT DNA, Coralville, Iowa). The sequences of the forward and reverse primer used are ATGGTCATGGTAAGTGGCA and CCTCTGCCATTGTCTAGCTT, respectively. Both male and female Knockout animals showed reduced expression of the ERα gene in the cardiac tissue as shown in Figure 1.

### Animal Studies

Wild type (ERα^flox/flox^) and cardiomyocyte-specific ERα knockout (cs-ERα^−/−^) male and female mice of C57BL/6 phenotype were bred and maintained at Washington University School of Medicine animal facility. Mice had free access to regular diet and water. At approximately 18–20 weeks of age, four mice from each group (total of 16 mice) were sacrificed after drawing blood for biochemical analysis, and their hearts were isolated and flash frozen for further analysis. Serum was separated by centrifugation from the collected blood samples for measurement of non-esterified fatty acids (NEFA), triglycerides (TG) and cholesterol concentrations. All experiments were performed in compliance with the guidelines for the care and use of research animals established by Animal Studies Committee of Washington University in St. Louis School of Medicine.

### Biochemical analysis

Serum NEFA, TG, and cholesterol concentrations were determined by using commercial kits for NEFA (Wako Diagnostics, VA), TG (Infinity Triglycerides Reagent, Thermo Scientific, USA), and cholesterol (Infinity total cholesterol, Thermo Scientific, USA). These assays were performed by the Diabetes Research Center (DRC) at Washington University School of Medicine. Blood glucose concentrations were measured by using commercial blood test strips (Accu-Check, Roche, USA) by using a drop of tail blood.

### Echocardiogram

Echocardiograms were performed using non-invasive ultrasound imaging with the Vevo2100 Ultrasound System (Visual Sonics Inc., Toronto, Ontario, Canada) at 18–20 weeks of age as described previously [19]. Briefly, mice were anesthetized with Avertin (2% solution, 0.05 mg/g body weight, IP) and secured to the imaging platform. Complete 2-dimensional, M-mode, and Doppler examinations using a 30 MHz transducer were performed to quantify left ventricular structure as well as diastolic and systolic function. All dimensional measurements were indexed to body weight.

### Microarray analysis

Total RNA was extracted from ∼50 mg of pulverized cardiac tissues using Qiagen Universal RNA isolation kit (Qiagen, Frederick, MD) following the manufacturer’s recommended protocol. On-column DNase removal was performed and total RNA concentration and purity were measured by absorbance ratio at 260 nm and 280 nm. Total RNA quality was then determined by Agilent 2100 bio analyzer (Agilent Technologies) according to the manufacturer’s recommendations. All samples used in the study had a RNA integrity number (RIN) number of 7 or above. RNA amplification, hybridization, and detection were performed at the Genome Technology Access Center, Washington University in Saint Louis. RNA transcripts were amplified by T7 linear amplification (Message Amp Total Prep amplification kit; Life Technologies). For reverse transcription, 400 ng of total cellular RNA sample (11 µl) was mixed with an oligo-dT T7 primer (1 µl), reaction buffer (2 µl, 10x), dNTP mix (4 µl), RNase Inhibitor (1 µl), and Arrayscript RT enzyme (1 µl) and then incubated at 42°C for 2 h. After a three minute incubation on ice, the cDNA underwent second strand synthesis after addition of water (63 µl), 10x second strand buffer (10 µl), dNTP mix (4 µl), DNA polymerase (2 µl), and RNase H (1 µl). This cocktail was incubated at 16°C for two hours. Following a column cleanup using Zymo DNA Clean and Concentrator 5s (Zymo Research) according to the manufacturer’s protocol, in vitro-transcription (IVT) was carried out by adding 10x T7 reaction buffer (2.5 µl), T7 biotin-NTP mix (2.5 µl), and T7 RNA polymerase enzyme mix (2.5 µl) and then incubated at 37°C. The IVT reaction was carried out for 14 hours. Following reaction termination with water (75 µl), the amplified RNAs (aRNA) were cleaned with RNA columns provided in the MessageAmp TotalPrep kit. The aRNAs were then quantitated on a spectrophotometer, and quality determined by the Agilent 2100 bio analyzer (Agilent Technologies) according to the manufacturer’s recommendations. 750 µg of each aRNA in water (5 µl) was suspended in Illumina “HYB” buffer (10 µl), heated to 65°C for five minutes, and allowed to cool to room temperature. The samples were applied to Illumina Mouse Ref-8v2 Expression BeadChips and hybridized at 58°C for 16–20 hours at high humidity. Arrays were washed according to Illumina standard protocol. Immobilized, biotinylated aRNAs were then detected by staining with cy3 streptavidin (1 µg cy3-SA per 1 ml of Illumina “Block E1”) for 10 minutes at room temperature. Arrays were washed and dried according to Illumina standard protocol. Arrays were scanned on an Illumina BeadArray Reader. Laser power and PMT voltage were kept constant for Cy3 scans. Images were quantitated by Illumina Beadscan, v3.

### Statistics and data analysis

Structural parameters from ECHO were normalized to body weight prior to statistical analysis. Echo and substrate data were analyzed using a 2-Way ANOVA model (Sex, Genotype, Sex*Genotype). Microarray data were imported into Illumina Genome Studio software. On-array spot replicates were averaged by Genome Studio and individual spot probe was reported. The bead chip data were normalized using the cubic spline method in the Illumina Genome Studio software package and exported in log_2_ metric. Raw and normalized data sets for all samples involved have been submitted to the National Center for Biotechnology Information Gene Expression Omnibus (GEO) repository under the accession number GSE55936.

**Figure 2:**
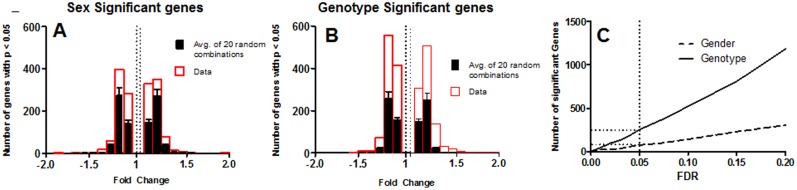
Statistical analysis and validation: Histogram for the number of significant genes and their fold changes from the actual combination of Bead Chips compared to 20 random combinations (p<0.05) are shown for both (A) sex and (B) Genotype. This shows the number of significantly identified genes in the actual combination is higher than number of false positives expected, even at low fold change. Number of significant genes as a function of FDR value is shown, at every FDR level genotypic variation is larger than the (C) sex variation. When corrected for multiple comparison (FDR) no significant fold changes are identified in any of the random combination until FDR = 0.20. Significant genes were calculated using Partek Genomics Suite, using three way ANOVA model on filtered dataset for signal intensity and batch effect (13, 155 probe sets of 25179) and p value <0.05, corrected for FDR <0.05.

A replicated (n = 4) 2^2^ factorial design was used to investigate the patterns of differentially expressed genes between sexes, genotypes, and any interactions between these two factors (male and female mice of wild type and cs-ERα^−/−^genotypes). The 16 samples were divided between two Illumina Mouse Ref-8v2 Expression Bead Chips in a pattern sufficient to identify and correct for batch effects, if any, between chips. Probe sets were filtered from the data set if fewer than 3 replicates in any group had detection p-values greater than 0.05. The filtered data were imported into the statistical program Partek Genomics Suite v6.6 for analyses. Initially, a three-way ANOVA model with interaction (sex, genotype, batch and sex*genotype) was applied to identify possible batch effects and if necessary to adjust the data accordingly. The 3-way ANOVA model was reapplied to the batch adjusted data to determine statistical differences, and this analysis was followed by contrast comparisons using Fisher Least Squares difference to determine the statistical significance. Fold changes between groups were calculated as the antilog of the log ratio of the two groups of interest (i.e. KO/WT, female/male). It was expected that the statistical noise (frequency of false positives) would be greater at low fold changes. Therefore, to confirm the sensitivity of the analysis with respect to fold change, the analysis was repeated on 20 different random combinations of the 16 Bead Chips. The distribution of fold changes for probe sets with a p-value <0.05 from the real combinations are plotted in Figure 2A (between sex) and 2B (between genotype) along with the average number obtained from the 20 random combinations. This analysis indicates that, even for fold changes as low as +/−1.1; the number of probe sets identified in the actual combination is higher than that expected by chance alone. The false discovery rate method of Benjamin and Hochberg was applied to the p-values from the ANOVA and contrast analysis. The number of statistically significant genes as a function of false discovery rate (FDR) level is shown in Figure 2C for both the genotype and sex differences. The purpose of this analysis was to determine a FDR level that would minimize the number of false positives at low fold change level and still provide a sufficient number of hits for downstream analysis. As seen in Figure 2C, an FDR of 0.05, the conventionally accepted level, identifies 208 genes with a genotype difference and 56 genes with sex differences. Although application of an FDR = 0.05 reduces the number of positive hits in the true comparison, the analysis totally eliminates false positive hits in the random combinations as depicted in Figure 2A & 2B.

### Gene Ontology (GO) and Pathway Analyses

For exploratory analysis, Hierarchical clustering (HCL) of the significant genes was carried out using Partek genomics suite. HCL analysis was performed on intensity data derived from the ANOVA analysis yielding 254 and 74 probe sets that are differentially expressed between genotype and sex, respectively. The fold changes were subject to normalization, shifting genes to mean of zero and standard deviation of one prior to clustering. Clustering was performed with complete linkage where the distance between two clusters is equal to the distance between the two furthest members of those clusters. The results from clustering analysis are provided in the Figure S1 and S2.

#### Gene Ontology (GO) Analysis

The significant gene lists, both for genotype and sex variations, were analyzed using Metacore. Analysis of the significant probe sets revealed that there were 208 and 56 identifiable unique genes that were different between genotype and sex, respectively. The gene set thus identified at the FDR 0.05 level was used to identify GO pathways and processes. GO was primarily used to probe the cellular localization of genes, the processes in which they are involved, the metabolic networks, and their potential pathways.

#### Pathway Analysis

To investigate the biological functions of the differentially expressed genes, pathway analysis was conducted using MetaCore. Analysis was performed using the 208 genes that were different between the knockout and wild type mice and for the 56 genes that were different between sexes. The dataset containing gene identifiers and corresponding expression values were uploaded on to the web portal and the identifiers were mapped to its corresponding object in MetaCore’s knowledgebase. Networks for the identified and mapped molecules were then algorithmically generated based on their connectivity. As our interests were primarily on the modulation of genomic actions by ERα, we conducted enrichment analysis for pathways and built network for transcription factors and receptors for the gene list. The transcription factor network analysis generates a list of transcription factors that have targets among the uploaded gene list, and the receptor network generates a list for which ligands are present in the uploaded data set. It then draws the shortest paths between these lists. The networks were generated and scored.

FDR threshold has been argued to be too restrictive in conducting pathway analysis [24]. Therefore, to gain a broader insight on altered pathways, we have relaxed FDR thresholding. We used the Compare Experiment Workflow module within MetaCore to compare variations in gene expression between male knockout mice to female knockout mice, normalized to their respective controls. The data identified the intersection network nodes on Metacore’s various ontologies from the two sexes.

## Results and Discussion

**Figure 3:**
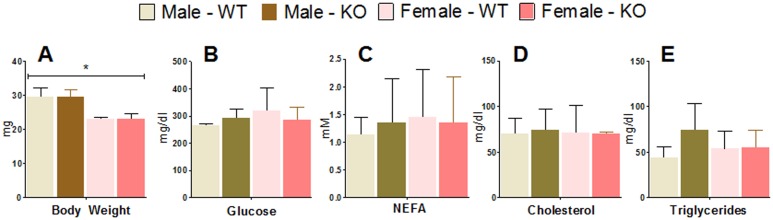
Weights and Serum substrate levels of wild type and cs-ERα−/− . (A) Weight of mice at the time of study ranging 18–20wks. Serum substrate concentrations of (B) Glucose, (C) NEFA, (D) Cholesterol and (E) Triglyceride were determined by spectroscopic analysis. Data are represented as mean ± SD (N = 4 for each category). *indicates sex difference P<0.05, Two-Way ANOVA.

In this work, we report on the generation of mice with cardiomyocyte-specific deletion of ERα (cs-ERα^−/−^ mice). In contrast to whole-body ERα knockout mice, which are obese [12] and exhibit significantly increased serum substrates (e.g., triglycerides, cholesterol, glucose) [13], cs-ERα^−/−^ mice were viable, overtly normal, and did not display significant variations in serum substrate concentrations nor weight (Figure 3). This observation underscores the significance of using an animal model with cardiac specific knockout of ERα in place of whole-body ERα knockout to investigate the role of ERα in the heart since the latter can result in cardio-metabolic remodeling due to systemic effects of ERα.

To characterize the role of ERα in the heart at baseline, we assessed differences in cardiac structure and function as well as performed microarray gene expression profiling on cs-ERα^−/−^ in comparison WT mice. Two–way ANOVA of echocardiographic measurements revealed that with the exception of the peak velocity of systolic mitral annular motion (S’), there were no significant genotypic variations in the remaining functional parameters. However, sex differences were observed in multiple structural parameters, such as, left ventricle posterior wall diameter, the left ventricle internal dimension, and the inter-ventricular septum (Table 1).

**Table 1:** Echocardiographic measurements.

	Male WT	Female WT	Male cs-ERα^−/−^	Female cs-ERα^−/−^	Stats
***Structural***					
LVPWId (mm/g)	0.03±0.003	0.04±0.002	0.03±0.006	0.039±0.003	a
LVPWIs (mm/g)	0.05±0.002	0.06±0.003	0.05±0.008	0.061±0.009	a
LVIDId (mm/g)	0.12±0.002	0.15±0.011	0.11±0.008	0.132±0.002	a, b
LVIDIs (mm/g)	0.06±0.005	0.08±0.010	0.006±0.005	0.067±0.003	a, b
IVSd	0.870±0.06	0.890±0.04	0.890±0.1401	0.940±0.064	a
IVSs	1.420±0.15	1.430±0.092	1.450±0.134	1.510±0.256	a
LVM	112.63±20.23	107.65±17.18	95.74±12.60	92.04±6.16	
LVMI (mg/g)	3.790±0.45	4.660±0.69	3.250±0.45	3.980±0.09	a, b
***Functional***					
FS (%)	48.68±3.16	47.01±3.47	45.80±2.91	48.88±2.44	
E’ (mm/s)	42.41±7.77	36.78±4.78	33.16±5.45	41.08±9.90	
A’ (mm/s)	33.16±6.22	N/A	N/A	N/A	
S’	34.63±1.44	28.17±3.10	26.84±2.98	28.32±4.10	b, c
IVCT (ms)	5.80±2.05	6.96±2.72	7.78±1.43	7.49±1.74	
ET	38.05±3.66	42.11±2.26	38.33±4.18	41.86±3.52	
IVRT (ms)	11.52±3.65	10.20±1.61	12.67±2.20	11.57±1.78	
Tei Index	0.45±0.09	0.41±0.10	0.53±0.04	0.46±0.08	

All dimensional measurements were indexed to body weight. FS, fractional shortening; E, peak velocity of early diastolic trans-mitral flow; A, peak velocity of late (atria) diastolic trans-mitral flow; S’, peak velocity of systolic mitral annular motion; E’, peak velocity of early diastolic mitral annular motion; A’, peak velocity of late (atrial) diastolic mitral annular motion; IVCT, iso-volumic contraction time; ET, LV ejection time; IVRT, iso-volumic relaxation time; Tei Index, LV performance index calculated as (IVCT+IVRT)/ET. Values given as mean ± SD (N = 4/group). N/A, not available; ^a^denotes sex significance between male and female mice; ^b^denotes genotypic significance between wild type and cs-ERα^−/−^ mice; ^c^Interaction term of ANOVA analysis was significant; P<0.05 was considered significant.

**Figure 4:**
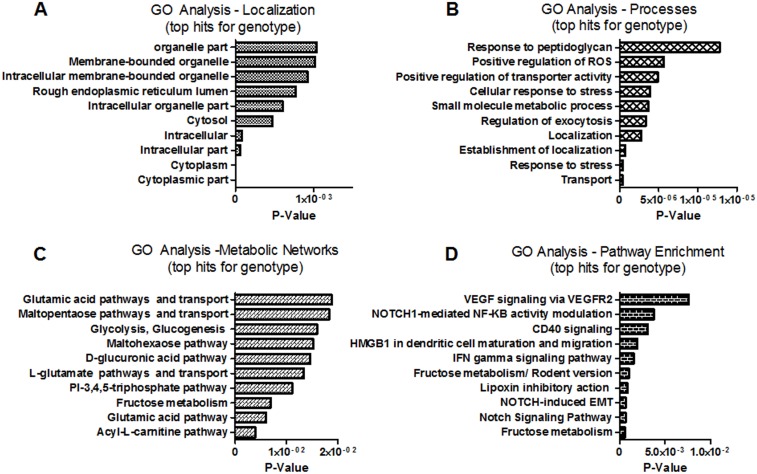
GO Enrichment Analysis for top hits of genotype significant Genes: GO Term analysis were performed using GeneGo on the list of significant genes varying by genotype, FDR <0.05. Top 10 hits (where applicable) is presented for (A) Localization, (B) Cellular Processes, (C) Metabolic Networks, and (D) Pathway Enrichment. P values for GO analysis are calculated based on hypergeometric distribution. The P-values on the graph indicate the probability of mapping of an experiment to a process to arise by chance.

**Figure 5:**
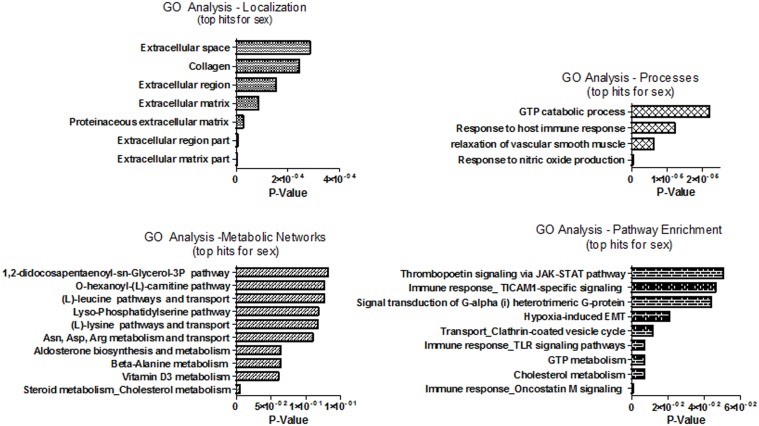
GO Enrichment Analysis for top hits of sex significant Genes: GO Term analysis were performed using MetaCore from GeneGo on the list of significant genes varying by sex, FDR <0.05. Top 10 hits (where applicable) are presented for (A) Localization, (B) Cellular Processes, (C) Metabolic Networks, and (D) Pathway Enrichment. P values are calculated based on hypergeometric distribution. The P-values on the graph indicate the probability of mapping of an experiment to a process to arise by chance.

Microarray analysis was performed to characterize variations in gene expression pattern attributed “genotype” and “sex”. ANOVA analysis of the microarray data with a FDR = 0.05 identified 208 genotype-specific genes and 56 sex-specific genes whose expression levels were significantly altered between cs-ERα^−/−^and WT mice (Table 1 and 2). With the exception of 8 genes, there was no overlap between the panel of genotypic- and sex- specific genes. The sensitivity of our analysis was confirmed by randomization of the dataset as explained in the methods section and is shown in Figure 2. To characterize these differences, GO analysis was carried out to identify gene-product differences in cellular localization, processes, and metabolic networks. The GO analysis suggested that gene-product differences linked to the genotype are distinct from those linked to the sex difference (Figure 4 and 5). In addition, pathway enrichment analysis was performed to delineate the pathways in which ERα participates, either directly or indirectly.

**Table 2:** List of significant, differentially expressed genes, attributed to genotype (P<0.05, 2-Way ANOVA) between ER Alpha KO and Wild type mice.

Gene Name	Symbol	FC	p.value
RNA binding protein, fox-1 homolog	A2bp1	−1.16	1.87E-04
4-aminobutyrate aminotransferase	Abat	1.35	3.27E-04
ATP-binding cassette, sub-family A (ABC1), member 8	Abca8a	1.17	7.17E-04
ATP-binding cassette, sub-family B (MDR/TAP), member 8	Abcb8	−1.16	1.82E-04
Acyl-CoA dehydrogenase, C-2 to C-3 short chain	Acads	−1.16	3.48E-04
Acyl-CoA thioesterase 7	Acot7	−1.19	1.07E-05
Acyl-CoA synthetase medium-chain family member 5	Acsm5	−1.41	3.29E-04
Aminoacylase 1	Acy1	−1.12	9.22E-04
Adenylosuccinate synthase like 1	Adssl1	1.19	4.11E-05
Aldo-keto reductase family 1 member B3	Akr1b3	1.29	6.88E-07
Aldolase B, fructose-bisphosphate	Aldob	−1.81	2.66E-05
Amylase, alpha 1	Amy1	−1.25	8.52E-05
Ankyrin repeat domain 1 (cardiac muscle)	Ankrd1	1.67	2.55E-05
Anoctamin 10	Ano10	−1.64	1.12E-04
Amyloid beta (A4) precursor protein-binding, family B, member 1 (Fe65)	Apbb1	−1.23	1.28E-04
Asialoglycoprotein receptor 2	Asgr2	−1.18	1.63E-04
Aspartate beta-hydroxylase	Asph	1.34	2.29E-05
ATPase, Ca++ transporting, cardiac muscle, fast twitch 1	Atp2a1	2.11	1.20E-04
ATPase, H+ transporting V0 subunit e2	Atp6v0e2	1.43	4.74E-04
ATPase, H+ transporting, lysosomal 34kDa, V1 subunit D	Atp6v1d	1.14	2.04E-04
Ataxin 1	Atxn1	−1.21	4.03E-04
B9 protein domain 2	B9d2	−1.17	5.87E-04
Bardet-Biedl syndrome 7	Bbs7	1.16	9.53E-04
Branched chain keto acid dehydrogenase E1, alpha polypeptide	Bckdha	−1.19	3.92E-05
B-cell CLL/lymphoma 7A	Bcl7a	−1.18	7.05E-04
Complement component 1, q subcomponent, B chain	C1qb	1.30	7.75E-04
Coiled-coil domain containing 80	Ccdc80	1.26	1.68E-05
Chemokine (C-C motif) ligand 27	Ccl27	1.19	2.90E-04
Cyclin G1	Ccng1	1.09	7.59E-04
CD164 molecule, sialomucin	Cd164	1.09	9.66E-05
CD84 molecule	Cd84	1.24	1.60E-04
CD86 molecule	Cd86	1.25	5.93E-04
complement factor properdin	Cfp	1.31	6.86E-04
CKLF-like MARVEL transmembrane domain containing 8	Cmtm8	−1.17	6.83E-05
Cordon-bleu protein-like 1	Cobll1	−1.21	2.13E-04
Coenzyme Q5 homolog, methyltransferase (S. cerevisiae)	Coq5	−1.10	1.90E-04
COX19 cytochrome c oxidase assembly homolog (S. cerevisiae)	Cox19	1.21	3.18E-06
Cysteine-rich protein 2	Crip2	−1.05	3.21E-04
Catenin (cadherin-associated protein), alpha 3	Ctnna3	−1.44	4.72E-07
CTP synthase 1	Ctps	1.15	5.77E-07
Cathepsin Z	Ctsz	1.22	1.67E-04
Dephospho-CoA kinase domain containing	Dcakd	−1.52	3.97E-08
Dynactin 2 (p50)	Dctn2	1.11	1.28E-04
DENN/MADD domain containing 2A	Dennd2a	−1.64	7.54E-09
DnaJ (Hsp40) homolog, subfamily C, member 30	Dnajc30	−1.05	3.37E-04
Destrin (actin depolymerizing factor)	Dstn	1.17	4.39E-04
Epoxide hydrolase 1, microsomal (xenobiotic)	Ephx1	1.26	4.88E-05
Electron-transfer-flavoprotein, beta polypeptide	Etfb	−1.10	7.49E-04
Exocyst complex component 2	Exoc2	−1.52	5.20E-07
Exocyst complex component 4	Exoc4	1.22	3.29E-05
Coagulation factor XIII, A1 polypeptide	F13a1	1.35	7.57E-04
Fatty acid desaturase 1	Fads1	1.22	3.66E-04
Fumarylacetoacetate hydrolase (fumarylacetoacetase)	Fah	−1.21	7.70E-04
Fumarylacetoacetate hydrolase domain containing 1	Fahd1	1.15	1.05E-04
Family with sequence similarity 122B	Fam122b	1.20	8.26E-05
F-box and leucine-rich repeat protein 12	Fbxl12	−1.14	8.47E-05
Fc fragment of IgG, low affinity IIIb, receptor (CD16b)	Fcgr3	1.23	3.20E-04
Fer (fms/fps related) protein kinase, testis specific 2	Fert2	−1.11	4.35E-04
Flt3-interacting zinc finger protein	Fiz1	−1.11	5.29E-06
FK506 binding protein 4, 59kDa	Fkbp4	−1.10	1.47E-04
Fibronectin type III domain containing 5	Fndc5	−1.18	7.91E-04
Formyl peptide receptor 2	Fpr2	1.36	6.51E-04
Follistatin-like 4	Fstl4	1.52	7.53E-05
Frataxin	Fxn	−1.13	7.21E-04
growth arrest-specific 6	Gas6	1.13	4.80E-05
MTOR associated protein, LST8 homolog	Gbl	−1.15	1.74E-04
Growth differentiation factor 15	Gdf15	1.73	9.05E-05
Glycerophosphodiester phosphodiesterase domain containing 1	Gdpd1	1.16	2.68E-04
Glucose-fructose oxidoreductase domain containing 1	Gfod1	−1.16	6.74E-05
Glycolipid transfer protein	Gltp	1.18	1.47E-04
Guanine nucleotide binding protein-like 1	Gnal1	−1.09	8.67E-04
Golgi SNAP receptor complex member 2	Gosr2	−1.14	1.69E-05
G protein-coupled receptor 34	Gpr34	1.32	2.48E-04
Trans-2,3-enoyl-CoA reductase	Gpsn2	−1.14	8.57E-04
Glyoxylate reductase/hydroxypyruvate reductase	Grhpr	1.14	2.72E-05
Glutamate receptor, ionotropic, N-methyl D-aspartate-associated protein 1 (glutamate binding)	Grina	1.08	3.02E-04
G protein-coupled receptor kinase 5	Grk5	1.35	7.11E-04
GTF2I repeat domain containing 2	Gtf2ird2	−1.13	4.75E-04
Guanylate kinase 1	Guk1	1.14	7.13E-05
High density lipoprotein binding protein	Hdlbp	−1.17	7.39E-05
HIG1 hypoxia inducible domain family, member 1B	Higd1b	−1.23	4.71E-04
Histone cluster 1, H2be	Hist1h2be	1.19	4.92E-04
Histone cluster 1, H2bk	Hist1h2bk	1.13	5.06E-04
Histone cluster 1, H3f	Hist1h3f	1.25	3.37E-04
Histone cluster 2, H3b	Hist2h3b	1.15	7.65E-04
High mobility group nucleosomal binding domain 2	Hmgn2	−1.15	9.83E-06
Hematological and neurological expressed 1	Hn1	1.44	1.93E-06
Heat shock 27kDa protein 1	Hspb1	1.09	8.78E-04
Islet cell autoantigen 1, 69kDa	Ica1	−1.10	7.54E-04
Interferon-induced protein with tetratricopeptide repeats 2	Ifit2	1.23	1.37E-04
Interleukin 13 receptor, alpha 1	Il13ra1	1.12	2.90E-04
Interleukin 15	Il15	−1.24	2.63E-04
Interleukin 28 receptor, alpha (interferon, lambda receptor)	Il28ra	1.18	4.94E-04
IMP2 inner mitochondrial membrane peptidase-like	Immp2l	−1.63	1.97E-10
Insulin-like 6	Insl6	1.27	2.35E-04
Importin 13	Ipo13	−1.23	6.57E-04
IQ motif containing GTPase activating protein 2	Iqgap2	1.23	2.53E-04
Potassium voltage-gated channel, Isk-related family, member 1	Kcne1	−1.25	7.06E-05
Potassium channel, subfamily V, member 2	Kcnv2	−1.47	2.12E-05
Lysosomal protein transmembrane 5	Laptm5	1.26	1.82E-04
Lipocalin 2	Lcn2	1.75	8.75E-05
Lectin, galactoside-binding, soluble, 3	Lgals3	2.08	2.05E-07
Lysozyme	Lyz	1.38	9.78E-05
Lysozyme 2	Lyz2	1.59	2.60E-06
Membrane associated guanylate kinase, WW and PDZ domain containing 3	Magi3	1.29	7.62E-04
MAGI family member, X-linked	Magix	−1.19	4.79E-04
Mitogen-activated protein kinase kinase kinase 3	Map3k3	−1.08	4.28E-04
Mitogen-activated protein kinase 11	Mapk11	−1.15	5.09E-04
Methyltransferase like 17	Mett11d1	−1.18	5.63E-04
Monoglyceride lipase	Mgll	−1.12	6.07E-04
Matrix metallopeptidase 23B	Mmp23	1.21	6.32E-04
Mannose receptor, C type 1	Mrc1	1.32	7.27E-06
Mitochondrial ribosomal protein S9	Mrps9	−1.15	1.10E-04
Myosin binding protein C, fast type	Mybpc2	1.72	4.45E-07
NCK-associated protein 1-like	Nckap1l	1.29	6.74E-05
Niemann-Pick disease, type C2	Npc2	1.15	6.35E-04
Natriuretic peptide A	Nppa	2.10	1.37E-05
Nuclear receptor binding protein 2	Nrbp2	1.28	1.06E-04
Nucleotide binding protein 1	Nubp1	1.26	2.33E-05
Nudix (nucleoside diphosphate linked moiety X)-type motif 5	Nudt5	1.19	8.72E-05
Oxysterol binding protein-like 3	Osbpl3	1.27	1.15E-04
Organic solute transporter alpha	Osta	1.47	1.98E-07
Prolyl 4-hydroxylase, beta polypeptide	P4hb	1.21	2.27E-04
Pantothenate kinase 3	Pank3	−1.20	3.59E-04
Polyamine oxidase (exo-N4-amino)	Paox	1.19	8.89E-04
Poly (ADP-ribose) polymerase family, member 12	Parp12	−1.45	2.67E-06
Polycomb group ring finger 6	Pcgf6	1.12	6.88E-04
Pyruvate dehydrogenase kinase, isozyme 1	Pdk1	−1.19	7.92E-05
Pyruvate dehydrogenase kinase, isozyme 2	Pdk2	−1.16	1.15E-04
Phosphofructokinase, platelet	Pfkp	1.34	4.65E-07
Pleckstrin homology-like domain, family A, member 3	Phlda3	1.60	7.20E-08
Paired-Ig-like receptor A4	Pira4	1.28	4.21E-05
Protein kinase (cAMP-dependent, catalytic) inhibitor alpha	Pkia	−1.10	9.53E-04
Plakophilin 2	Pkp2	1.16	5.05E-04
Phospholipase C, gamma 2 (phosphatidylinositol-specific)	Plcg2	1.27	5.66E-04
Pleckstrin homology domain containing, family A (phosphoinositide binding specific) member 8	Plekha8	1.18	7.06E-04
Protein kinase C, delta	Prkcd	1.24	5.12E-07
Prolactin receptor	Prlr	−1.35	3.99E-06
Protein arginine methyltransferase 2	Prmt2	1.19	2.88E-04
Prion protein-interacting protein1	Prnpip1	−1.17	9.65E-04
Phosphoserine aminotransferase 1	Psat1	1.25	5.77E-04
Prostaglandin D2 synthase 21kDa (brain)	Ptgds	1.48	2.15E-04
Protein tyrosine phosphatase-like (proline instead of catalytic arginine), member A	Ptpla	−1.14	5.96E-04
Protein tyrosine phosphatase, receptor type, O	Ptpro	1.33	5.63E-04
Polymerase I and transcript release factor	Ptrf	−1.21	8.29E-04
RAB31, member RAS oncogene family	Rab31	1.27	3.87E-05
RAB3D, member RAS oncogene family	Rab3d	1.17	6.39E-04
RAB, member of RAS oncogene family-like 3	Rabl3	−1.11	3.71E-04
RAP2B, member of RAS oncogene family	Rap2b	1.16	5.28E-04
RNA binding motif protein 38	Rbm38	−1.13	6.17E-04
RNA binding motif protein 47	Rbm47	1.20	8.04E-04
Rhomboid, veinlet-like 3 (Drosophila)	Rhbdl3	−1.19	1.07E-04
Ring finger protein 135	Rnf135	−1.15	3.49E-04
Ring finger protein 208	Rnf208	1.36	5.56E-04
Retinal outer segment membrane protein 1	Rom1	1.26	2.63E-05
Ribonuclease P/MRP 25kDa subunit	Rpp25	1.46	2.87E-07
S100 calcium binding protein A13	S100a13	1.25	5.25E-04
SH3-binding domain kinase 1	Sbk	−1.19	3.42E-04
Secretory carrier membrane protein 5	Scamp5	1.26	9.10E-04
SH3-domain binding protein 2	Sh3bp2	1.18	2.58E-04
SH3-binding domain protein 5-like	Sh3bp5l	1.11	1.71E-04
Src homology 2 domain containing transforming protein D	Shd	1.43	3.42E-06
Serine hydroxymethyltransferase 2 (mitochondrial)	Shmt2	−1.16	1.89E-05
Solute carrier family 17 (sodium-dependent inorganic phosphate cotransporter), member 7	Slc17a7	1.57	3.16E-05
Solute carrier family 19 (thiamine transporter), member 2	Slc19a2	1.29	1.58E-05
Solute carrier family 25, member 34	Slc25a34	−1.20	7.43E-04
Solute carrier family 35, member B4	Slc35b4	1.17	9.44E-05
Solute carrier family 39 (zinc transporter), member 13	Slc39a13	1.13	7.25E-04
Solute carrier family 45, member 2	Slc45a2	−1.13	5.25E-04
Solute carrier family 7 (orphan transporter), member 4	Slc7a4	1.17	4.59E-04
Single-strand-selective monofunctional uracil-DNA glycosylase 1	Smug1	−1.28	1.92E-04
Small nuclear RNA activating complex, polypeptide 4, 190kDa	Snapc4	1.13	9.39E-04
Single-stranded DNA binding protein 2	Ssbp2	−1.40	3.07E-04
Sushi, von Willebrand factor type A, EGF and pentraxin domain containing 1	Svep1	1.26	2.82E-04
Tachykinin, precursor 1	Tac1	−1.37	7.12E-04
TAF15 RNA polymerase II, TATA box binding protein (TBP)-associated factor, 68kDa	Taf15	−1.43	3.55E-04
Transcription elongation factor B (SIII), polypeptide 1 (15kDa, elongin C)	Tceb1	1.12	3.18E-04
Tescalcin	Tesc	−1.29	1.69E-04
Translocase of inner mitochondrial membrane 10 homolog (yeast)	Timm10	1.15	2.20E-06
TIMP metallopeptidase inhibitor 1	Timp1	2.81	7.81E-06
Toll-like receptor 2	Tlr2	1.25	3.46E-04
Transmembrane channel-like 7	Tmc7	1.14	6.57E-04
Transmembrane protein 141	Tmem141	1.11	3.90E-05
Transmembrane protein 164	Tmem164	−1.32	7.42E-05
Transmembrane protein 167A	Tmem167	1.19	9.09E-05
Transmembrane protein 176B	Tmem176b	1.33	1.36E-05
Transmembrane protein 38A	Tmem38a	−1.11	4.82E-04
Transmembrane protein 43	Tmem43	1.11	9.58E-04
Transmembrane protein 62	Tmem62	1.17	4.07E-04
Tumor protein D52	Tpd52	1.19	4.55E-05
Thiamin pyrophosphokinase 1	Tpk1	1.24	4.81E-04
Trafficking protein particle complex 2-like	Trappc2l	−1.11	1.74E-04
Tripartite motif containing 21	Trim21	−1.18	9.21E-04
TROVE domain family, member 2	Trove2	1.23	5.84E-04
Tetraspanin 12	Tspan12	1.25	4.27E-05
Tetraspanin 17	Tspan17	1.26	6.30E-05
Translocator protein (18kDa)	Tspo	1.17	4.39E-05
Tubulin tyrosine ligase-like family, member 1	Ttll1	−1.32	1.03E-06
Uracil-DNA glycosylase	Ung	−1.36	4.05E-04
Uridine phosphorylase 1	Upp1	1.27	1.27E-04
Uridine phosphorylase 2	Upp2	−1.13	6.90E-04
Ubiquinol-cytochrome c reductase core protein I	Uqcrc1	−1.08	9.37E-04
Ubiquitin specific peptidase 16	Usp16	1.21	6.76E-04
Vacuolar protein sorting 29 homolog	Vps29	1.13	6.37E-05
WNT1 inducible signaling pathway protein 2	Wisp2	1.38	4.74E-04
Yes-associated protein 1	Yap1	−1.14	5.88E-04
Zinc finger, DHHC-type containing 12	Zdhhc12	1.21	5.14E-04
Zinc finger protein 398	Zfp398	−1.22	4.71E-04

### Differentially expressed genes attributed to genotype

Fold changes in the expression of 208 genes that were significant for genotypic variations were imported into MetaCore for GO enrichment analysis. The top 10 (where applicable) networks and pathways from this analysis are shown in Figure 4. Figure 4A shows that the absence of ERα largely affects expression of genes encoding membrane bound proteins and proteins localized in the intracellular lumen. The processes in which these gene products are involved range from regulation of reactive oxygen species (ROS), stress response, to metabolism of small molecules (Figure 4B). Our analyses indicated that expression of enzymes involved in metabolism of carbohydrates and their intermediates and acyl-L-carnitine pathway were affected by genotype (Figure 4C). The pathway enrichment analysis indicated that multiple Notch1-regulated signaling pathways are affected in cs-ERα^−/−^mice (Figure 4D). The network of genes included those that encode phosphofructokinase, amylase, aldose reductase, and aldolase (Table 2 and 3).

**Table 3:** List of significant, differentially expressed genes, attributed to sex (P<0.05, 2-Way ANOVA) between cs-ERα^−/−^ and Wild type mice.

Gene Name	Symbol	FC	p.value
Branched chain amino-acid transaminase 1, cytosolic	Bcat1	−1.20	3.09E-05
Fumarylacetoacetate hydrolase (fumarylacetoacetase)	Fah	−1.26	1.77E-04
Glycosyltransferase 8 domain containing	Glt8d1	1.14	1.34E-04
Immunoglobulin superfamily, member 1	Igsf1	−1.32	1.08E-04
Cytochrome P450, family 27, subfamily A, polypeptide	Cyp27a1	−1.20	1.60E-04
Guanylate cyclase 1, soluble, alpha 3	Gucy1a3	1.37	1.57E-04
Hydroxyacylglutathione hydrolase-like	Haghl	−1.21	1.94E-04
Coenzyme Q5 homolog, methyltransferase	Coq5	−1.11	9.35E-05
Eotaxin	Ccl11	−1.75	1.02E-05
Bone morphogenetic protein-binding endothelial cell precursor-derived regulator	Bmper	−1.38	1.68E-04
Hydroxysteroid (11-beta) dehydrogenase 1	Hsd11b1	-2.40	1.50E-08
Collagen, type XIV, alpha	Col14a1	1.31	6.32E-05
DEAD (Asp-Glu-Ala-Asp) box polypeptide 3, Y-linked	Ddx3y	−16.45	7.96E-16
DIRAS family, GTP-binding RAS-like	Diras2	1.20	1.91E-04
Chemokine (C-X-C motif) ligand	Cxcl14	−1.68	1.42E-06
DnaJ (Hsp40) homolog, subfamily C, member 30	Dnajc30	−1.07	1.13E-05
Eukaryotic translation elongation factor 1 delta	Eef1d	1.10	1.46E-04
Eukaryotic translation initiation factor 2, subunit 3 gamma, 52kDa pseudogene	Eif2s3x	1.59	3.08E-07
Eukaryotic translation initiation factor 2, subunit 3 gamma, 52kDa pseudogene	Eif2s3y	-9.75	9.63E-14
Eukaryotic translation initiation factor 4E family member 3	Eif4e3	−1.14	1.13E-04
Elastin microfibril interfacer 2	Emilin2	1.20	2.43E-04
Guanine nucleotide binding protein (G protein), gamma 8	Gng8	1.33	2.33E-04
Golgi SNAP receptor complex member 2	Gosr2	−1.12	8.56E-05
G protein-coupled receptor associated sorting protein 1	Gprasp1	1.22	1.50E-04
Indolethylamine N-methyltransferase	Inmt	-2.23	8.35E-05
Potassium voltage-gated channel, Isk-related family, member 1	Kcne1	2.32	1.04E-04
Kinesin family member 3C	Kif3c	1.14	1.18E-04
Kallikrein 1-related petidase b26	Klk1b26	−1.34	1.46E-04
Lysyl oxidase	Lox	−1.40	2.20E-04
Keratan sulfate proteoglycan lumican	Lum	1.48	1.94E-04
Myeloid differentiation protein-2	Ly96	−1.06	9.58E-05
Microspherule protein 1	Mcrs1	1.12	2.60E-04
Midkine (neurite growth-promoting factor 2)	Mdk	1.52	2.64E-06
Nidogen 1	Nid1	−1.20	1.17E-04
Organic solute transporter alpha	Osta	1.27	2.45E-05
Oviductal glycoprotein 1, 120kDa	Ovgp1	1.22	2.55E-05
Pleiotrophin	Ptn	1.78	3.82E-08
RAS-like, family 10, member B	Rasl10b	−1.27	5.37E-05
Ring finger protein 219	Rnf219	1.19	2.55E-05
Serpin peptidase inhibitor, clade A	Serpina3n	-2.57	1.60E-04
Solute carrier family 1 (glial high affinity glutamate transporter), member 3	Slc1a3	1.26	2.54E-04
Slowmo homolog 2 (Drosophila)	Slmo2	−1.10	1.38E-04
Sortilin 1	Sort1	1.22	6.28E-05
Vesicle-associated membrane protein 7	Sybl1	1.43	1.82E-06
Epicardin	Tcf21	1.27	2.55E-04
Tudor domain-containing protein 2	Tdrkh	1.21	2.57E-04
Transmembrane protein 141	Tmem141	−1.10	1.07E-04
Transmembrane protein 201	Tmem201	1.17	2.41E-05
Transmembrane protein 38A	Tmem38a	−1.14	9.36E-05
Transmembrane protein 82	Tmem82	1.35	5.97E-05
TruB pseudouridine (psi) synthase homolog 2	Trub2	−1.16	1.42E-04
Ubiquitin specific peptidase 18	Usp18	1.26	7.17E-05
Histone demethylase UTX	Utx	1.82	5.11E-07
Vesicle-associated membrane protein 4	Vamp4	1.14	1.37E-04
WNT1 inducible signaling pathway protein 2	Wisp2	-2.03	3.17E-07
X (inactive)-specific transcript (non-protein coding)	Xist	2.23	2.35E-08

**Figure 6:**
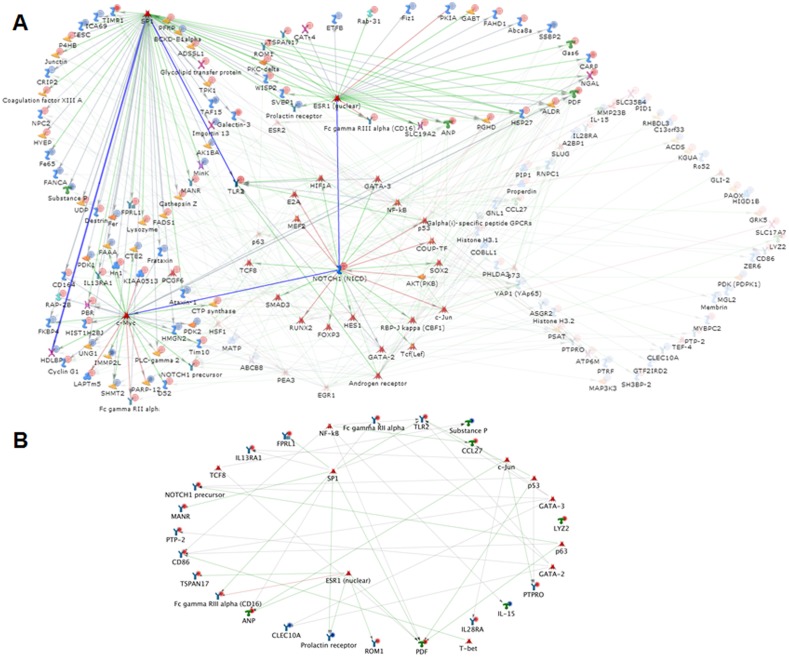
Network analysis of genes significant for genotype: List of significant genes (root list) varying by genotype was uploaded on to GeneGo portal and network built. Transcription factor network (A) was drawn using analyze network function for transcription factors. In this analysis for every transcription factor with direct ligand (s) in the root list, the algorithm generates a sub-network consisting of all shortest paths from that transcription factor to the closest receptor with direct target (s) in the root list. Receptor networks (B) was drawn using build network for your data option. Except receptors and receptor ligands, others were hidden from this network in order to visualize the alteration in receptor networks. The figure shows the differentially expressed genes in the network (Blue for down-regulated and Red for up-regulated).

ERα has been shown to be indispensable for glucose uptake in mouse heart [11]. Whole body inactivation of ERα results in obesity, insulin resistance, and glucose intolerance [12], [13], [25]. Alteration in Glut4 expression due to variations in ERα has been implicated in insulin resistance and subsequent glucose intolerance in mice [26]. SP1 and NFκB are key modulators of Glut4, a prominent glucose transporter [25], [27]. In agreement with these data, the gene network from our results is largely regulated by three transcription factors; Sp1, Notch 1, and C-Myc (Figure 6A). Furthermore, we observed that NFκB, along with Sp1, is involved in regulation of the genes controlling insulin utilization that are differentially expressed between cs-ERα^−/−^and WT mice. Though estrogen activated expression and translocation of GLUT4 has been shown to be vital for glucose disposal, our microarray analysis failed to reveal any significant difference in Glut4 expression between knockout and wild type animals. However, we observe up regulation of Rab31, a member of the Ras oncogene family, which is known to modulate glucose homeostasis by alterations in GLUT4 partitioning between the cell membrane and intracellular vesicles (Table 2). Similar observations were made in mouse hearts from whole body ERα KO where there was no significant changes in mRNA or protein levels of these transporters, but rather alterations in glucose transport due to variation in GLUT4 partitioning [28].

In addition to alterations in carbohydrate metabolism, we observed that the deletion of ERα affects expression of genes involved in lipid metabolism (Table 2). GO analysis of differentially expressed genes in cs-ERα^−/−^mice reveals differences in expression of enzymes involved in acylcarnitine metabolism (Figure 3C). ERα-mediated regulation of lipogenic genes is well documented [29], [30]. For example, observed variations in desaturase enzyme mRNA expression (Table 2) and activity have been implicated in altering insulin sensitivity in whole body ERα knockout [31], [32].

### Core regulators of genes that vary by genotype

Because ERα is known to have significant regulatory roles in transcriptional modulation and receptor mediated signaling, network analysis was performed to identify differentially expressed networks between cs-ERα^−/−^and WT mice. Our analysis suggests that the transcription factors Stimulating protein 1 (SP1), Estrogen Receptor Alpha (ERα), and C-Myc are at the hub of transcriptional modulation connecting most of the significant differentially expressed genes (Figure 6A). More than seventy-five genes were differentially expressed among these three networks, with nearly one-third being down-regulated and two-thirds being up-regulated in cs-ERα^−/−^ mice. In addition, thirteen genes coding for receptors and five genes coding for receptor ligands were differentially expressed in the cs-ERα^−/−^ mice (Figure 6B). All of the genes coding for receptors were up-regulated in cs-ERα^−/−^ mice, with the exception of the genes coding for prolactin receptor and C-type lectin receptor Clec10a. The up-regulated genes include a G protein-coupled receptor (GPCR) type receptor; interleukin 13 receptor alpha 1 (Il13ra1); Notch 1 precursor; toll-like receptor 2 (Tlr2); and interleukin 28 receptor alpha (Il28ra), several of which have known roles in cardiac metabolism.

### Differentially expressed genes attributed to sex

The GO analysis based on sex is distinct from that of genotype. The primary sites of localization for sex significant gene-products are in the extracellular space and matrix (Figure 5A). The list of processes shown in Figure 5B suggests that, NOS signaling and processes involved in smooth muscle relaxation are affected the most, primarily due to variations in expression levels of the genes Gucy1a3, Emilin2, and Lum (Table 3). The metabolic network analysis indicates that the genes that are differentially expressed between male and female have roles in amino acid and steroid metabolism (Figure 5C). The genes that are significantly different include Bcat1, Eif2s3y, Fah, Inmt, and Haghl, which are all down regulated and Diras2, Lum, and Ovgp1, which are up-regulated (Table 3). Finally, GO pathway enrichment analysis indicates that signaling pathways with a primary role in immune response, such as the JAK-STAT pathway, TICAM signaling, TLR signaling, Oncostatin signaling (Figure 5D), are primarily affected.

### Core regulators of genes that vary by sex

**Figure 7:**
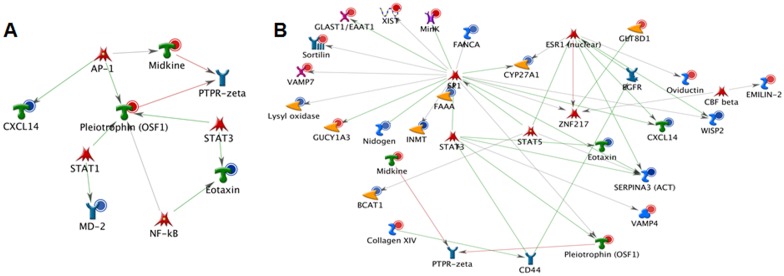
Network analysis of genes significant for sex: List of significant genes (root list) varying by sex was uploaded on to GeneGo tool and network was built. Receptor networks (A) was drawn using build network for your data option. Except receptors and receptor ligands, others were hidden from this network in order to visualize the alteration in receptor networks.. Transcription factor network (B) was drawn using analyze network function for transcription factors. In this analysis for every transcription factor with direct ligand (s) in the root list, the algorithm generates a sub-network consisting of all shortest paths from that transcription factor to the closest receptor with direct target (s) in the root list. The figure shows the differentially expressed genes in the network (Blue for down-regulated and Red for up-regulated).

Network analysis based on sex difference presents a smaller network of receptor genes compared to genotypic differences. Only one receptor coding gene, Ly96 (MD-2), is down-regulated in females. However, there are 4 receptor ligand encoding genes that are differentially expressed between female and male mice. In females, Pf4 (Cxcl4) and Ccl11 (Eotaxin) are down-regulated whereas Mdk (Midkine) and Ptn (Pleiotrophin) are up-regulated (Figure 7A). As observed in our analysis between genotypes (previous section), we find that Stat3, Stat5, Znf, Sp1 and Esr1 (ERα are at the core connecting a larger proportion of these differentially expressed genes (Figure 7B).

### Novel candidates for ERα mediated regulation of cardiac function

**Figure 8:**
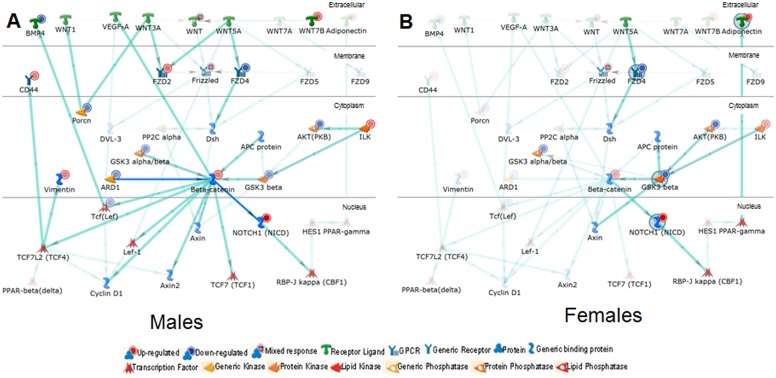
Alteration in Wnt signaling in cs-ERα^−/−^mice: Variations in WNT signaling in males (A) and females (B) in cs-ERα^−/−^mice as identified through microarray profiling and subsequent network mapping using MetaCore. The network is filtered for cardiac tissue in mice and in a layout based on sub-cellular localization from top to bottom. The traced and bolded network components represent the modified genes and the genes that are connected to them highlighting the difference observed in cs-ERα^−/−^ mice of both sex.

Table 4 lists novel significant (FDR <0.05) genes that are directly regulated by ERα. While additional studies are needed to characterize their role in regulating cardiac function and biology, in-depth analysis of the genes and their networks may provide understanding of the role of ERα in the heart. For example, we observe that WNT1 inducible signaling pathway protein 2 (Wisp2) is differentially expressed and is directly under regulation of ERα. While Wnt/β-catenin signaling, both canonical and non-canonical, has been implicated in cardiac function [33]–[35], there has been no direct evidence for the cross talk between these two key pathways (ERα and Wnt/β-catenin). As mentioned earlier in methods section, to understand how Wnt Signaling may be affected by the absence of ERα, we relaxed the FDR thresholding and subjected the data to Compare Experiment Workflow in MetaCore. Our analysis suggests that there are sex differences in the Wnt/β-catenin signaling pathway. We observe that several key effectors of the WNT canonical signaling pathway such as frizzled receptor (Fzd), β-catenin, GSK- 3β, Tcf, and Sfrp1 are differentially affected in male and female ERα^−/−^ mice (i.e. both sex and genotype differences are observed) (Figure 8 A, B). Similar effects of ERα regulation of Wnt, β-catenin signaling pathways have been reported in. the uterus by Hewitt et al [36]. Taken together, additional studies are needed to fully characterize the interplay and impact of ERα with Wnt/β-catenin signaling pathway, among others, in cardiac function.

**Table 4:** List of significantly different genes (P<0.05, 2-Way ANOVA) between cs-ERα^−/−^ and Wild type mice and networked directly to ERα.

Gene Name	Symbol	FC	p.value	
ATP-binding cassette, sub-family A (ABC1), member 8	Abca8a	1.17	7.17E-04	
Aldo-keto reductase family 1 member B3	Akr1b3	1.29	6.88E-07	
Ankyrin repeat domain 1 (cardiac muscle)	Ankrd1	1.67	2.55E-05	
Electron-transfer-flavoprotein, beta polypeptide	Etfb	−1.10	7.49E-04	
Fc fragment of IgG, low affinity IIIb, receptor (CD16b)	Fcgr3	1.23	3.20E-04	
Fumarylacetoacetate hydrolase domain containing 1	Fahd1	1.15	1.05E-04	
Flt3-interacting zinc finger protein	Fiz1	−1.11	5.29E-06	
growth arrest-specific 6	Gas6	1.13	4.80E-05	
Growth differentiation factor 15	Gdf15	1.73	9.05E-05	
Heat shock 27kDa protein 1	Hspb1	1.09	8.78E-04	
Lipocalin 2	Lcn2	1.75	8.75E-05	
Natriuretic peptide A	Nppa	2.10	1.37E-05	
Protein kinase (cAMP-dependent, catalytic) inhibitor alpha	Pkia	−1.10	9.53E-04	
Protein kinase C, delta	Prkcd	1.24	5.12E-07	
Prolactin receptor	Prlr	−1.35	3.99E-06	
Prostaglandin D2 synthase 21kDa (brain)	Ptgds	1.48	2.15E-04	
RAB31, member RAS oncogene family	Rab31	1.27	3.87E-05	
Retinal outer segment membrane protein 1	Rom1	1.26	2.63E-05	
Solute carrier family 19 (thiamine transporter), member 2	Slc19a2	1.29	1.58E-05	
Solute carrier family 7 (orphan transporter), member 4	Slc7a4	1.17	4.59E-04	
Single-stranded DNA binding protein 2	Ssbp2	−1.40	3.07E-04	
Sushi, von Willebrand factor type A, EGF and pentraxin domain containing 1	Svep1	1.26	2.82E-04	
Tetraspanin 17	Tspan17	1.26	6.30E-05	
WNT1 inducible signaling pathway protein 2	Wisp2	1.38	4.74E-04	

## Conclusion

In conclusion, cs-ERα^−/−^ mice raised under normal diet in absence of external stress were viable and overtly normal. Our data suggests that ERα modulates numerous genes in the heart that are involved in transcriptional regulation, metabolic control, and oxidative stress in a sex-specific manner. This study has identified potential networks through which ERα may affect cardiac biology. Our study also identified novel genes that are potentially under direct regulation by ERα and whose role in the heart is yet unclear, and we have shown how their biological relevance can be identified through Wnt signaling. It is likely that alterations in the dietary pattern, aging, or induction of metabolic stress could reveal additional phenotypic and metabolic differences for which further studies could lead to a better understanding of cardiac biology and potentially, improved treatment in a sex-dependent manner.

## Supporting Information

Figure S1 **Hierarchical Cluster analysis of genotype significant genes from cardiac mRNA microarray results.** mRNA expression patterns were established for cardiac tissue from both male and female, ERα ^−/−^ and wild type mice. HCL analysis was performed on intensity data using partek genomic suites for the significant gene list. FDR (0.05) corrected genes with a p value of 0.05 or less were considered significant.
(DOCX)

Figure S2 **Hierarchical Cluster analysis of sex significant genes from cardiac mRNA microarray results.** mRNA expression patterns were established for cardiac tissue from both male and female, ERα ^−/−^ and wild type mice. HCL analysis was performed on intensity data using partek genomics suite for the significant gene list. FDR (0.05) corrected genes with a p value of 0.05 or less were considered significant.
(DOCX)
