# Supplementary material for: An Animal Model with a Cardiomyocyte-Specific Deletion of Estrogen Receptor Alpha: Functional, Metabolic, and Differential Network Analysis
Source: PLoS One. 2014 Jul 7;9(7):e101900. doi: 10.1371/journal.pone.0101900 (PMC4085004; doi:10.1371/journal.pone.0101900)
Supplement: Figure S1 — Hierarchical Cluster analysis of genotype significant genes from cardiac mRNA microarray results. mRNA expression patterns were established for cardiac tissue from both male and female, ERα −/− and wild type mice. HCL analysis was performed on intensity data using partek genomic suites for the significant gene list. FDR (0.05) corrected genes with a p value of 0.05 or less were considered significant. (DOCX) [file pone.0101900.s001.docx]

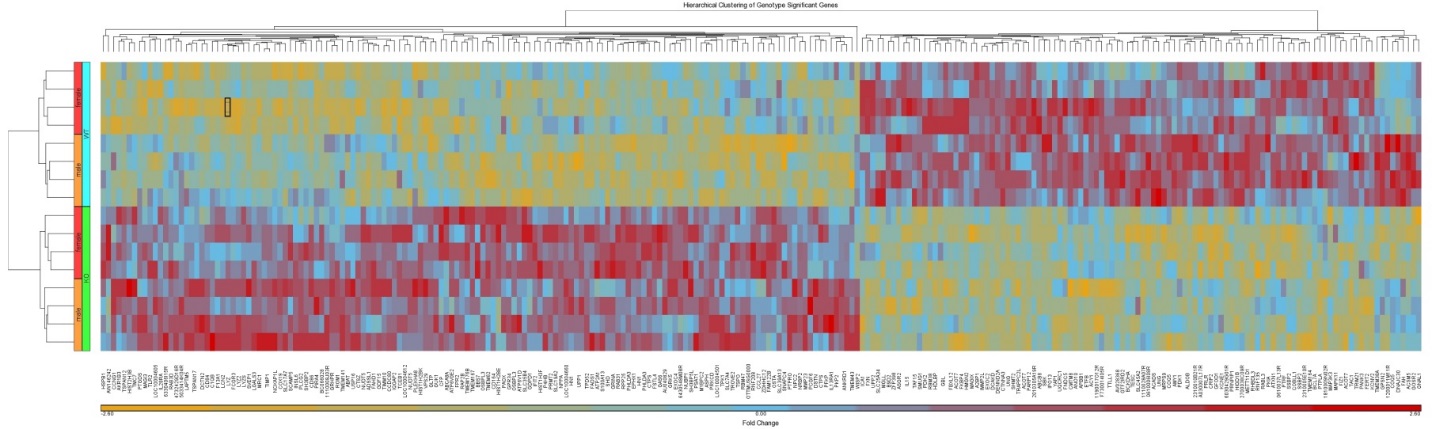


**Figure S1**

Hierarchical Cluster analysis of genotype significant genes from cardiac mRNA microarray results. mRNA expression patterns were established for cardiac tissue from both male and female, ERα ^-/-^ and wild type mice. HCL analysis was performed on intensity data using partek genomic suites for the significant gene list. FDR (0.05) corrected genes with a p value of 0.05 or less were considered significant.
